# Mediastinal Mass Caused by Granulomatous Reaction to Foreign Plant Material Following a Spontaneous Esophageal Rupture

**DOI:** 10.7759/cureus.26828

**Published:** 2022-07-13

**Authors:** Mohammad Darweesh, Susan Kullab, Mahmoud M Mansour, Ratib Mahfouz, Adham E Obeidat

**Affiliations:** 1 Internal Medicine, East Tennessee State University, Johnson City, USA; 2 Internal Medicine, University of Missouri School of Medicine, Columbia, USA; 3 Internal Medicine, Kent Hospital/Brown University, Warwick, USA; 4 Internal Medicine, University of Hawaiʻi, Honolulu, USA

**Keywords:** plant material, esophageal rupture, granulomatous reaction, anterior mediastinal mass, mediastinal mass

## Abstract

The discovery of a mediastinal mass presents a wide array of differential diagnoses which largely depends on the boundaries of the mass and its contents. Both computed tomography (CT) and magnetic resonance imaging (MRI) of the chest can determine radiologic compartmentalization to aid in diagnosis. Tissue biopsy for pathology, however, is necessary for final diagnosis. The establishment of a diagnosis should not be delayed, as mediastinal mass may be due to serious causes such as malignancy or infection. Here, we present a rare case of a 72-year-old male with a mediastinal mass that formed as a complication of traumatic esophageal perforation during cardiac arrest. Pathology revealed foreign plant material with granuloma formation secondary to food residue as the etiology of the mass.

## Introduction

Diagnosis of a mediastinal mass can present a challenging feat. Thymoma, teratoma, thyroid goiter, and lymphoma are common causes; however, the differential diagnosis is quite broad [1]. Determining the etiology of a mediastinal mass begins with identifying its location within the mediastinum. The International Thymic Malignancy Interest Group (ITMIG) has established boundaries to classify these masses into three categories: prevascular, visceral, and paravertebral [2]. Classification may further be divided by the contents of the mass, such as tissue, fluid, fat, or vascular structure. Chest CT or MRI is used to classify mediastinal mass into these categories and aid in diagnosis. Tissue biopsy or complete removal of the mass is then performed, depending on the structure and location. It is essential to establish a diagnosis, as causes such as malignancy and infection may be fatal. Furthermore, enlarging masses can be detrimental if they compress on structures such as the trachea or superior vena cava.

Despite being relatively uncommon, esophageal perforation carries high morbidity and mortality that can reach up to 40% [3]. The symptoms at presentation can mimic other serious thoracic conditions, such as myocardial infarction, which adds to the delay in diagnosis. The most common cause of esophageal perforation is iatrogenic at sites of luminal narrowing during interventions [4]. Depending on the age of presentation, the extent of the perforation, and the underlying pathology of the esophagus, the optimal treatment for esophageal perforation varies [3].

## Case presentation

A 72-year-old male with a past medical history significant for hypertension, gastroesophageal reflux disease, and nutcracker esophagus presented to the emergency department (ED) with a chief complaint of chest pain. He had a recent history of cardiac arrest post a choking episode two months earlier. He underwent chest CT at that time, which showed pneumomediastinum and pneumothorax with left lobar pneumonia and left-sided pleural effusion. He underwent thoracotomy and laparotomy with the repair of lower esophageal rupture. The empyema was drained, and food material was recovered from both the mediastinum and pleural cavity during surgery. A gastrostomy tube was placed for nutrition. He was treated with multiple courses of intravenous antibiotics. He recovered well and was discharged to a rehabilitation facility.

**Figure 1:**
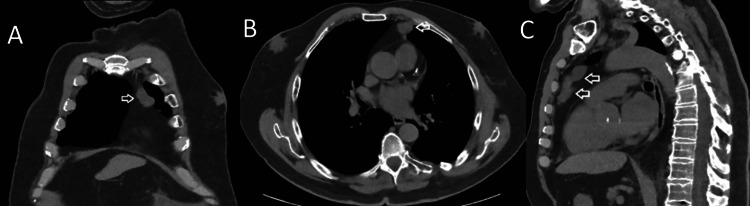
CT chest without contrast showing a new 2.2 x 1.7 x 4.5 cm soft tissue mass in the anterior mediastinum (white arrows) A: Coronal section, B: axial section, and C: sagittal section.

Two months later, the patient returned to the ED with chest pain. He described the pain as sharp and pleuritic, located at the left posterior chest wall with local tenderness to palpation. He denied fever, chills, shortness of breath, cough, weight loss, or night sweats. Physical examination was remarkable for tenderness at the left lower posterior chest. His incision site was well-healed. He was afebrile and hemodynamically stable. Initial laboratory studies did not reveal any abnormalities. He underwent a chest CT without contrast that showed left lateral mildly displaced subacute fifth through ninth rib fractures, as well as an unexpected new soft tissue mass in the anterior mediastinum measuring 2.2 x 1.7 x 4.5 cm as shown in Figures 1A-1C. The soft tissue mass was presumed to be an abscess.

**Figure 2:**
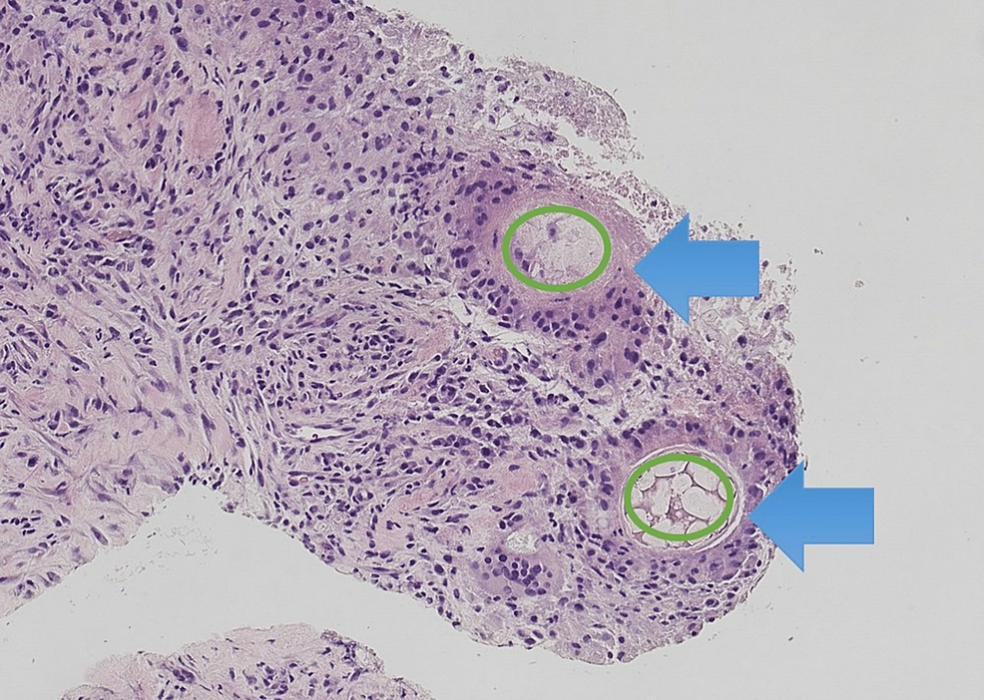
Plant foreign material (green circles) with granulomatous reaction (blue arrows) Hematoxylin and eosin stain (H&E), 20X.

**Figure 3:**
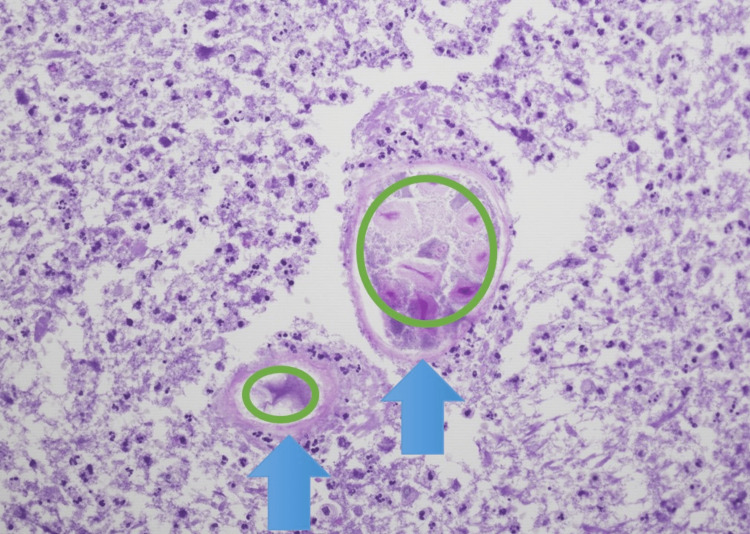
Plant foreign material (green circles) with granulomatous reaction (blue arrows) Hematoxylin and eosin stain (H&E), 40X.

**Figure 4:**
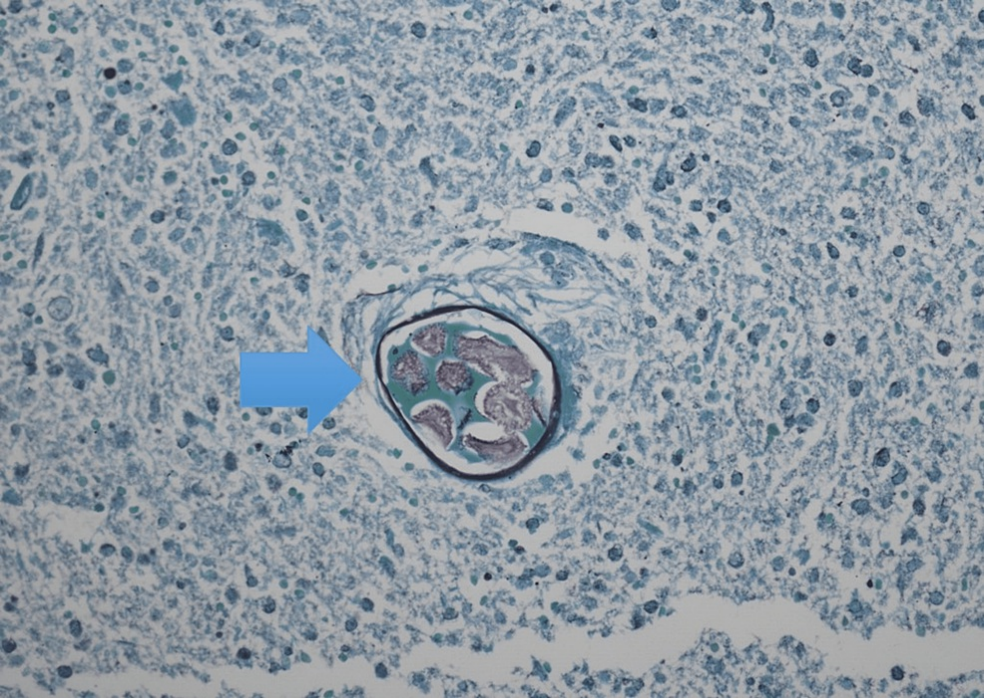
Yeast form of fungal organisms (blue arrow) GMS stain, 20X. GMS: Grocott methenamine silver.

Initially, the patient was treated with broad-spectrum antibiotics. He did not improve; therefore, a CT-guided aspiration of the mass was performed. No significant lymphoid immunophenotypic abnormalities appeared on flow cytometry. The sample was predominantly granulocytic cells with very few lymphocytes detected. Cultures grew *Candida albicans*. Gross examination of the aspirate showed partially necrotic plant material associated with reactive fibrous tissue with infiltrates of chronic inflammatory cells. Pathology showed extensive necrosis with chronic inflammation, giant cells, and plant foreign material with a granulomatous reaction that represented food residues (Figures 2, 3). Grocott methenamine silver (GMS) stain was positive for the yeast form of a fungal organism (Figure 4). The patient was prescribed a prolonged course of fluconazole and amoxicillin/clavulanic acid with clinical and radiological improvement on follow-up.

## Discussion

Esophageal perforation is a fatal condition if not diagnosed in a timely manner. It carries high morbidity and mortality. With iatrogenic perforation secondary to endoscopy and luminal narrowing being the most common, other etiologies also include spontaneous trauma and caustic substance ingestion [3,4]. Although spontaneous esophageal rupture also called Boerhaave syndrome (BS) can occur in patients with a normal esophagus, several underlying conditions usually predispose them to perforation such as malignancies, achalasia, gastroesophageal reflux disease (GERD), strictures, and others. In our case, the patient presented initially with spontaneous esophageal rupture which occurred suddenly after having severe vomiting in the setting of his known nutcracker esophagus. BS usually classically presents with severe retching and vomiting preceding the onset of pain [5], with pain being the most common symptom [4]. The physical examination can help in 27% of cases that usually demonstrated cervical or mediastinal emphysema [6]; this was not the case for our patient whose physical exam was initially unremarkable.

The most common cause of death in patients with esophageal rupture is sepsis leading to multiorgan failure; the most frequently reported complications include persistent leak, fistula formation, mediastinitis, empyema, esophageal stricture, pneumonia, abscess, mediastinitis, and sepsis [7-11]. No description in the literature for delayed complications of esophageal rupture makes this case the first of its kind to describe such a complication. Anatomically, the esophagus is situated in the posterior mediastinum; therefore, esophageal tumors or cysts typically present as posterior mediastinal or visceral masses. In this patient’s case, the mediastinal mass, which consisted of food particles most likely resulting from previous traumatic esophageal perforation, was peculiarly located in the anterior mediastinum.

It is highly unusual for food to travel to the anterior mediastinum rather than remain close to the esophagus. This is thought to be due to chronic inflammation and organization leading to anterior migration of the granuloma or less likely secondary to food escaping during surgery to the anterior part of the chest. Upon a review of medical literature, reported esophageal repair complications included persistent leakage, prolonged mechanical ventilation, pneumonia, arrhythmia, status asthmaticus, and empyema [11]. Mediastinal abscesses were also mentioned [11]. However, a mediastinal mass in the anterior mediastinum without systemic symptoms of infection has not yet been reported in the medical literature.

## Conclusions

This case represents a novel etiology of anterior mediastinal mass as well as an unusual presentation of delayed complications of esophageal perforation. The plant foreign material originated from food that escaped from the perforated esophagus either during cardiac arrest or during the surgical esophageal repair. It is not well understood if the granuloma migrated or originated in the anterior mediastinum. Health care providers should always consider the less common causes of mediastinal masses, especially if patients do not improve with treatment. Thorough patient history is essential, as recent esophageal intervention can be a key factor in the differential diagnosis of mediastinal mass.
